# Strangles in Arabian horses in Egypt: Clinical, epidemiological, hematological, and biochemical aspects

**DOI:** 10.14202/vetworld.2016.820-826

**Published:** 2016-08-06

**Authors:** Ahmed N. F. Neamat-Allah, Hend M. El Damaty

**Affiliations:** 1 Department of Clinical Pathology, Faculty of Veterinary Medicine, Zagazig University, Alzeraa Street Postal Code 44511, Zagazig City, Sharkia Province, Egypt; 2 Department of Animal Medicine, Faculty of Veterinary Medicine, Zagazig University, Alzeraa Street Postal Code 44511, Zagazig City, Sharkia Province, Egypt

**Keywords:** biochemical, Egypt, hematology, horse, strangles, *Streptococcus equi*

## Abstract

**Aim:**

Respiratory tract infections are considered the major problem of equine worldwide. Strangles is an infectious and highly contagious respiratory bacterial disease of equine caused by *Streptococcus equi*. This study is aimed to evaluate some clinical and epidemiological investigation associated with strangles and to study the hematological and biochemical changes in 20 Arabian horse naturally infected with *S. equi* during the disease and after 10 days from treatment by procaine penicillin with benzathine penicillin.

**Materials and Methods:**

A total of 490 Arabian horses have been examined, 120 (24.5%) have been clinically diagnosed as strangles. Under complete aseptic conditions, nasal swabs and pus samples from those were collected for bacterial culture. 20 horses from the positive infected with *S. equi* have been treated by 6 mg/kg b.wt procaine penicillin with 4.5 mg/kg b.wt benzathine penicillin deep intramuscular injection/twice dose/4 days interval.

**Results:**

102 horses (20.8%) were found positive for *S. equi*. Horses with age group under 1 year were the most prone to strangles (32.25%) followed by horses of the age group from 1 to 2 years (20%) and finally of the age group over 2-4 years (11.89%). Hematological parameters revealed anemia in the infected horses, while leucogram revealed a significant increase in the total leucocytic, granulocytic and monocytic counts without a significant change in the lymphocytic count. Biochemical parameters revealed a significant increase in serum aspartate aminotransferase, total proteins, globulins, cardiac troponin I (cTnI), and potassium. In other side, hypoalbuminemia and hyponatremia have been reported, whereas alanine aminotransferase activity and creatinine level showed non-significant changes. Respiratory acidosis has been exhibited in the infected horses. Treatment of horses by procaine penicillin with benzathine penicillin revealed improvement of these parameters toward the healthy horses.

**Conclusion:**

*S. equi* easily spreads from infected to susceptible horses through contaminated water and other fomites. Therefore, good biosecurity is very important if the welfare and economic costs of an outbreak are to be reduced. The presence of respiratory acidosis with increased of cTnI could indicate pneumonia secondary to strangles with risk of heart involvement.

## Introduction

The Arabian horse is a versatile breed. Arabians dominate the discipline of endurance riding and compete today in many fields of equestrian activity. They consider one of the most famous top 10 popular horse breeds around the world. Nowadays, they are reared worldwide, but their land of origin is the Middle East [1]. Equines like any other domestic animals are very susceptible to a variety of infectious diseases [2]. Respiratory tract infections considered the major hazard for equine worldwide [3].

Strangles is an infectious respiratory bacterial disease of Equidae. It is considered one from the most 3 significant respiratory diseases of horses [4]. This infection caused by *Streptococcus equi* [5]. After an incubation period ranged from 3 days to 2 weeks from exposure to the causative agent, the infective organisms shed in nasal discharges and pus from opened lymph nodes [6]. The infected horse distinguished by fever, upper respiratory tract manifestations, dyspnea, and anorexia. Within 24 h of onset of fever, the sick horse develops bilateral, serous to mucoid nasal discharge which lately became mucopurulent. The occlusive effect of the lymph node enlargement is the source of the disease name “strangles,” which in late stages may resulted in suffocation. In Egypt, there are several outbreaks of strangles had been recorded in several horse breeding stations as well as individual horses have been reported [7].

Abscessed lymph node in head and neck of horse may be indicative to strangles, especially if animals of the same lot have similar clinical signs, however, bacteriological culture of nasal swabs, nasal washes, and aspirated pus from abscesses remains the gold standard [8,9].

Thus, our study was aimed to: (i) Studying the prevalence of strangles in Arabian horses, (ii) also hematological and biochemical analysis in 20 Arabian horses positive infected with *S. equi* during the disease and after 10 days from treatment by penicillin were done to evaluate the treatment therapy.

## Materials and Methods

### Ethical approval

This study has been done in accordance with the principles and guidelines of animal care and use with the help of veterinarians of the selected station.

### Arabian horse

A governmental station for Arabian horses located at Cairo province, Egypt was visited during the period from January 2015 to February 2016 to perform the epidemiological investigation of strangles. A total of 490 Arabian horses were used in this study. The station has been visited weekly for sampling of horses showing strangles suspected signs (strangled breathing with roaring sounds, abscessation of submaxillary, parotid, and retropharyngeal lymph nodes, hyperthermia, and anorexia with general debilitating conditions). Each horse underwent a thoroughly clinical examination as described by Radostits *et al*. [2], particularly the lymph nodes found in the head and neck region. Case history of each affected horse was collected and recorded.

### Sampling and bacterial isolation

**Figure-1:**
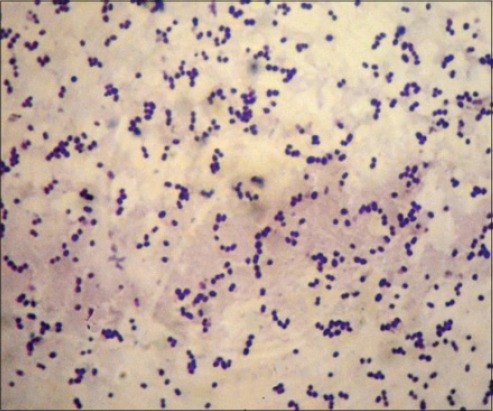
Microscopical examination of *Streptococcus equi* stained with Gram-stain showing Gram-positive cocci arranged in pairs and chains (Oil, 100×).

Nasal swabs and lymph nodes draining materials from the clinically affected horses [10] were immediately transported to the laboratory in an ice-cooled container for bacteriological examination at Faculty of Veterinary Medicine, Zagazig University. Samples were cultured on selective Edward media for *Streptococcus* and incubated at 37°C for 24 h under complete aerobic conditions. Beta-hemolytic colonies were subcultured on 5% blood agar [11]. Biochemical tests such as Gram-stain, catalase test and growth in 6.5% sodium chloride broth, and sugar fermentation were carried out [12]. *S*. *equi* appear as Gram-positive cocci in the form of pairs or chains (Figure-1), catalase test was negative and its inability to grow in 6.5% sodium chloride broth. Isolates identified as *S*. *equi* ferment the salicin and sucrose while, not ferment sorbitol, lactose, inulin, trehalose, raffinose, and mannitol [13]. *S. equi* is differentiated from *Streptococcus zooepidemicus* using the aspect that *S. zooepidemicus* could ferment sorbitol and lactose.

### Treatment

Around, 20 from the positive *S. equi* infected horses reared in the governmental station were treated by 6 mg/kg b.wt procaine penicillin with 4.5 mg/kg body weight benzathine penicillin equivalent to 1 ml per 25 kg b.wt deep intramuscular injection/twice dose/4 days interval [14].

### Blood samples

Blood samples have been collected during the disease and after 10 days from the treatment of the positive *S*. *equi* infected horses. Another 20 horses have been used as apparent healthy. Blood was taken into three sets. 1^st^ set of blood samples were taken into ethylenediaminetetraacetic acid tubes for determination of hematological parameters. Under anaerobic conditions, the 2^nd^ set of blood samples was collected in glass heparinized tubes for blood gas analysis. The 3^rd^ set of blood samples was taken into sterile test tubes without anticoagulant for separation of serum to be used later for analysis of biochemical parameters.

### Hematological studies

Full automatic digital cell counter (Hospitex Hemascreen 18, Italy) was used for determination of total erythrocytic count (red blood cells [RBCs]), hemoglobin (Hb), packed cell volume (PCV), total leucocytic count (TLC), neutrophil, eosinophil and basophil (GRA), monocytes and some eosinophil (MID), and lymphocytes (LYM).

### Serum biochemical studies

The serum has been separated by centrifugation at 3000 rpm for 25 min and stored at −20°C until used, then was tested spectrophotometrically for the biochemical parameters. Total serum proteins, albumin and globulins levels, activities of serum alanine and aspartate aminotransferase (ALT and AST), creatinine level, sodium (Na^+^) and potassium (K^+^), and cardiac troponin I (cTnI) were measured in full automated biochemistry analyzer (Chemray 240. USSR). Blood gas analyzers (ABL 5 Radiometer Copenhagen) have been used for estimation of pH, PCO_2_, PO_2_, and HCO_3_.

### Statistical analysis

Data of epidemiology of strangles in Arabian horses were analyzed using SPSS (v.16) Software. Chi-square analysis test was used to study the association between the prevalence rate of strangles and risk factors (age and season), and the results were considered to be highly significant at (< 0.01). The hematological and biochemical data were statistically analyzed using the one-way analysis of variance [15]. Means at the same row followed by different letters were significantly different, and the highest value was represented with the letter (a).

## Results and Discussion

**Figure-2:**
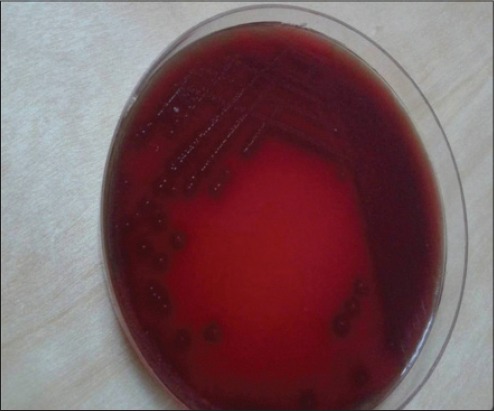
*Streptococcus equi* on blood agar media showing beta-hemolytic dew-drop like colonies.

**Figure-3:**
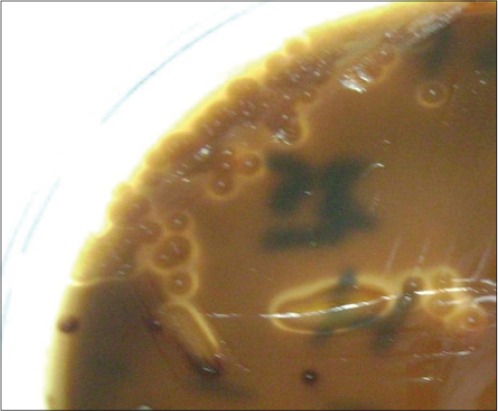
*Streptococcus equi* on Edward media showing beta-hemolytic dew-drop like colonies.

A total of 490 horses were examined, 120 nasal swabs and pus samples (24.48%) were collected from horses clinically diagnosed as strangles and examined bacteriologically. Beta-hemolytic, medium-sized, mucoid, dew-drop like colonies of *S*. *equi* on blood agar and Edward media (Figures-2 and 3) were recovered from 102 samples (20.8%), and 18 samples had non-*S. equi* isolates (3.67%). Therefore, at the animal level, the estimated prevalence of strangles was (24.48%), and at the isolated *S*. *equi* level, the estimated prevalence of strangles was (20.8%).

**Figure-4:**
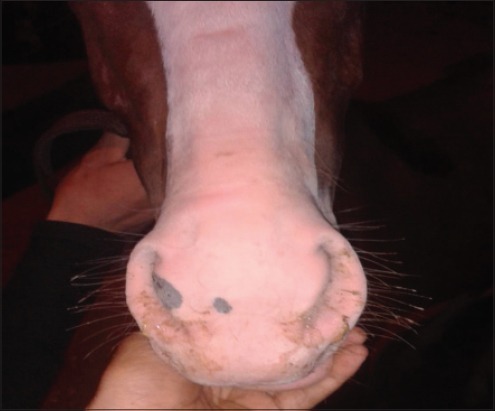
Mucopurulent nasal discharge originating from infected retropharyngeal lymph node in strangled Arabian horse.

**Figure-5:**
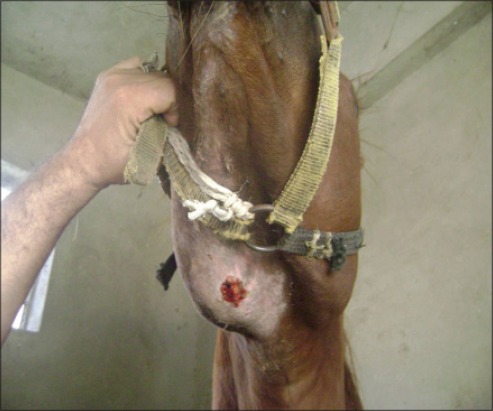
Strangled Arabian horse showing submaxillary lymph node abscess.

**Figure-6:**
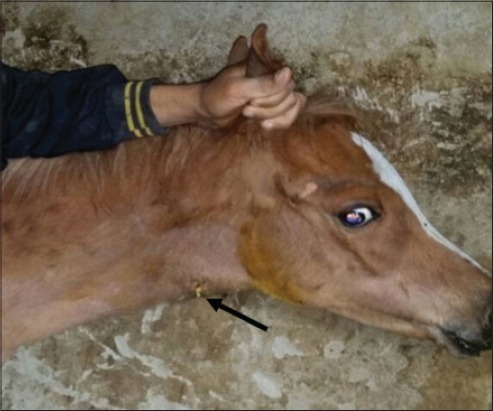
Horse suffering from strangles showing drained abscess of retropharyngeal lymph node (arrow).

**Figure-7:**
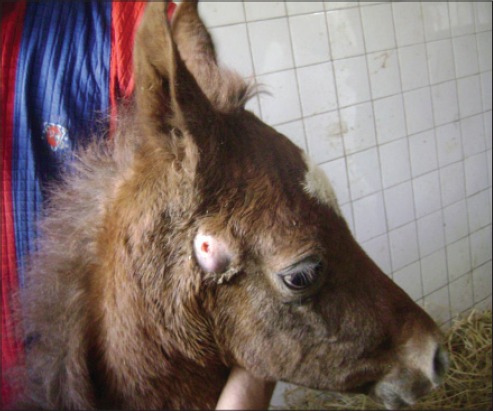
A foal suffering from strangles showing swollen parotid lymph node.

**Figure-8:**
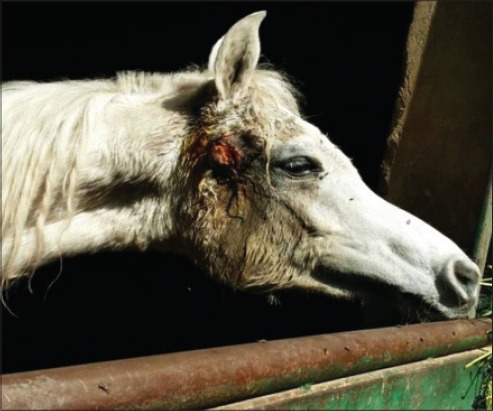
Swollen parotid lymph node with guttural pouch empyema in strangled horse.

The most common clinical symptoms appeared on the infected Arabian horses are fever, serous nasal discharge that later becomes mucopurulent (Figure-4) and even purulent, evidence of submaxillary, retropharyngeal, and parotid lymphadenopathy (Figures-5-7). Lymph nodes are initially firm but become fluctuant before rupturing at 7-10 days from the onset of clinical signs and guttural pouch empyema (Figure-8) that in agreement with Kvarka [16].

These findings are in agreement with the results of Helal and Hassan [7,17], in Egypt who found the prevalence rates of strangles in horse was 19.57% and 20.6%, respectively, and disagree with Ijaz *et al*. [18], who examined 250 horses and found that 113 (45.2%) horses positive for *S. equi*. A higher prevalence of strangles may be due to the presence of carrier horses that lead to spread and persistence of infection.

Many causes for failure to identify the presence of *S*. *equi* in clinical strangles cases that may be either due to low bacterial shedding according to the stage of the disease [2,13] or variation in method of sampling collection and/or chance of overgrowth of other bacteria particularly (*S*. *zooepidemicus*) [19]. On the other hand, as there is increasing evidence that *S*. *zooepidemicus* can also act as a contagious upper respiratory pathogen in horses [20].

The prevalence of strangles was found significant (p<0.05) among horses of the different age groups (Table-1). Horses in age group under 1 year of age are the most susceptible to develop strangles (32.25%), followed by horses of the age group from 1 to 2 years age (20%) and finally of the age group over 2-4 years age (11.89%). Our results are harmonious with Ijaz *et al*. [21], who proved that horses ≤2 years of age are most susceptible than adult these and also correlate with the findings of Manzoor *et al*. [22], who reported that horses at any ages could be infected by *S*. *equi*, but the disease is most common and severe in the younger age.

**Table-1:** Prevalence of strangles in different age groups of Arabian horses.

Age group (years)	Number of horses	Number of horses positive for *S. equi*	Percentage
<1	155	50	32.25
1-2	150	30	20
>2-4	185	22	11.89
Total	490	102	20.81

Chi-square analysis showed a significant effect on the prevalence of strangles among all age groups (Chi-square=121.076, p=0.002). *S. equi: Streptococcus equi*

The highest occurrence of strangles was increased significantly (p<0.01) among horses in the spring and autumn season 33.33% and 18.57%, respectively (Table 2), compared to 9 % in the winter and 17% in the summer seasons, this may be due to exposure to stress conditions as poor feeding, overcrowding, and relative humidity. Our results are consistent with Helal [7], who concluded that the occurrence of strangles in all seasons of the year but higher occurrence was found in the spring (45.52%). Similarly, our study also correlates with the findings of Manzoor *et al*. [22] and Ebid *et al*. [23], who recorded that highest prevalence of strangles was during the spring season, and disagree with Radostits *et al*. [2], who reported that strangles is mostly occurred in the cold and wet season.

**Table-2:** Prevalence of strangles among Arabian horses in different seasons of the year.

Season	Number of horses	Number of horses positive for *S. equi*	Percentage
Winter	100	9	9
Spring	150	50	33.33
Summer	140	26	18.57
Autumn	100	17	17
Total	490	102	20.81

Chi-square analysis showed a significant effect on the prevalence of strangles in different seasons (Chi-square=19.048, p=0.000). *S. equi: Streptococcus equi*

Hematological parameters revealed anemia in the strangled Arabian horses that represented by a significant reduction in erythrocytic counts, Hb concentrations, and PCV (Table-3). This could be due to block of iron release from reticuloendothelial storage during infection, thus became unavailable for utilization in Hb synthesis resulting in suppression of erythropoiesis [24]. These results are in agreement with those proved by Ismail *et al*. [25] and Mbengue *et al*. [26], who reported that strangled horses suffered from a significant decrease in RBCs, Hb, and PCV as compared with healthy horses. On the other side, TLC (Table-3) revealed a significant increase in TLC, GRA, and MID in strangled horses without change in LYM count. These results were similar to those obtained by Ijaz *et al*. [18], who reported pronounced leucocytosis with neutrophilia. In the same direction, our findings are in agreement with the results of Canfield *et al*. [27] and Ijaz *et al*. [28], who reported leucocytosis as outcomes of neutrophilia in experimentally infected horses with *S. equi*. These alterations were developed within two days of infection and in some horses may be continue to 35 days. Leucocytosis and neutrophilia commonly occur in horses in association with septic conditions. Septic inflammation is commonly associated with bacterial infection-like *S*. *equi* infections [29].

**Table-3:** Alteration in some hematological parameters in Arabian horses naturally infected with *S. equi* during the disease after 10 days from penicillin treatment compared with the healthy horses (mean values±SE).

Groups				
Parameters	Healthy Arabian horses	Strangled Arabian horses		

		Before treatment	After 10 days from start of penicillin treatment	F test
RBCs count (×10^6^/μl)	7.93^a^±0.04	7.24^c^±0.06	7.67^b^±0.02	**
Hb (g %)	12.26^a^±0.09	11.06^b^±0.17	12.06^a^±0.09	**
PCV (%)	35.62^a^±0.22	32.8^c^±0.46	34.52^b^±0.09	**
TLC (×10^3^/μl)	6.06^b^±0.16	9.86^a^±0.09	6.24^b^±0.21	**
LYM (×10^3^/μl)	1.90^a^±0.05	1.91^a^±0.04	1.88^a^±0.05	N.S
MID (×10^3^/μl)	1.46^b^±0.22	2.89^a^±0.25	1.64^b^±0.12	**
GRA (×10^3^/μ)	2.69^b^±0.07	5.05^a^±0.14	2.71^b^±0.05	**

Means in the same row with different superscript letters are significantly different.

** Highly significant difference at p≤0.01.

N.S=Not significant, RBCs=Total erythrocytic count, Hb=Hemoglobin concentration, PCV=Packed cell volume, RBCs=Total erythrocytic count, LYM=Lymphocytes, TLC=Total leucocytic count, GRA=Neutrophil, eosinophil, and basophil, MID=Monocytes and some eosinophil, SE=Standard error, *S. equi: Streptococcus equi*

Proteinogram (Table-4) showed a significant increase in the total proteins in strangles infected horse. This increase was due to hyperglobulinemia which characteristic for abscess formation [2]. The increase in the serum globulins level might be due to the increase in the immunoglobulins which indirectly reflect the serum total proteins levels. The significant decrease in the albumin level could be due to the lowering of feed intake [30]. Arabian horses infected with *S*. *equi* revealed a significant increase in serum AST activity that may be due to the local myositis which could attribute to an open abscess in the infected horse. Furthermore, this finding is in agreement with Dunnett *et al*. [31], who concluded that serum AST has been increased with muscle damage. Non-significant alteration in serum ALT reflects that *S*. *equi* infection did not effect on hepatic tissues of the Arabian horses.

**Table-4:** Alteration in some biochemical parameters in Arabian horses naturally infected with *S. equi* during the disease after 10 days from penicillin treatment compared with the healthy horses (mean values±SE).

Groups				
Parameters	Healthy Arabian horses	Strangled Arabian horses		

		Before treatment	After 10 days from start of penicillin treatment	F test
Total proteins (g/dl)	6.41^b^±0.08	6.88^a^±0.03	6.86^a^±0.06	**
Albumin (g/dl)	3.55^a^±0.03	2.91^c^±0.11	3.23^b^±0.15	**
Globulins (g/dl)	2.86^c^±0.07	3.97^a^±0.13	3.63^b^±0.07	**
ALT (Unit/L)	11.46^a^±0.81	10.90^a^±0.79	11.80^a^±0.86	N.S
AST (Unit/L)	295.2^b^±5.35	311.80^a^±3.30	305.60^ab^±2.37	*
Creatinine (mg/dl)	0.71^a^±0.07	0.68^a^±0.06	0.72^a^±0.05	N.S
Na (mEq/L)	135.50^a^±2.05	112.37^b^±2.21	134.96^a^±1.87	**
K (mEq/L)	5.42^b^±2.05	6.82^a^±2.05	5.29^b^±2.05	**
Troponin I (ng/ml)	0.038^b^±0.009	0.134^a^±0.01	0.062^b^±0.009	**

Means in the same row with different superscript letters are significantly different.

** Highly significant difference at p≤0.01,

* Significant difference at p≤0.05.

N.S=Not significant, ALT=Alanine aminotransferase, AST=Aspartate aminotransferase, Na=Sodium, K=Potassium, SE=Standard error, *S. equi: Streptococcus equi*

At the same time, non-significant change in creatinine level (Table-4) concluded that *S*. *equi* infection did not effect on renal tissue of the Arabian horses. In regard to serum electrolytes, the present work showed a significant decrease in serum sodium and increase in serum potassium levels in strangled horses. Hyperkalemia could be due to acidosis [32]. These results are in agreement with Ismail *et al*. [25], who reported hyperkalemia in respiratory diseased horses infected by *S*. *equi* which in turn leading to cardiac arrhythmias, and thus could be explaining the significant increase in the serum cTnI level in strangled horse which partially agree with Ali [33].

Acid-base results (Table-5) indicate the presence of respiratory acidosis; this clarified by the presence of a significant increase in PCO_2_ values associated with decrease in blood pH and PO_2_ without alteration in HCO_3_. Respiratory acidosis may be due to asphyxia as enlarged lymph nodes compressing on the larynx and or pneumonia as a secondary to *S. equi* infection [34,35]. The effect of acidosis is concerning to the respiratory system. The increased PCO_2_ tension of the blood and reduction of bicarbonate causes an increase of respiratory rate by stimulation of the respiratory center [2].

**Table-5:** Blood gasses and acid-base balance in Arabian horses naturally infected with *S. equi* during the disease after 10 days from penicillin treatment compared with the healthy horses (mean values±SE).

Groups				
Parameters	Healthy Arabian horses	Strangled Arabian horses		

		Before treatment	After 10 days from start of penicillin treatment	F test
pH	7.40^a^±0.03	6.83^b^±0.04	7.39^a^±0.02	**
PCO_2_ (mmHg)	39.8^b^±0.58	52.6^a^±0.92	40.6^b^±0.67	**
PO_2_ (mmHg)	28.54^a^±0.25	22.86^b^±1.29	28.68^a^±0.20	**
HCO_3_ (mEq/L)	23.84^a^±0.12	23.78^a^±0.10	23.71^a^±0.14	N.S

Means in the same row with different superscript letters are significantly different.

** Highly significant at p<0.01. PCO_2_=Partial pressure of carbon dioxide, PO_2_=Partial pressure of oxygen, HCO_3_=Bicarbonate, N.S=Not significant, *S. equi: Streptococcus equi*

After 10 days from the treatment of positive *S. equi* infected horses by recommended dose of 6 mg/kg b.wt procaine penicillin with 4.5 mg/kg b.wt benzathine penicillin revealed improvement of the hematological, biochemical, and acid-base values toward the parameters of healthy horses.

These results provide an insight into the potential hazard of *S. equi* infection in Arabian horses. However, we still lack in-depth studies to determine carrier animals. This ability of *S. equi* to establish persistent infection in carrier animals is likely to be responsible for the high prevalence of strangles around the world, and the identification of healthy carriers is the key to preventing new outbreaks of this disease.

## Conclusion

A higher occurrence of *S. equi* was recorded in the young foals compared to the adult one. *S. equi* easily spreads from infected to susceptible horses through contaminated water and other fomites. Therefore, good biosecurity is important if the welfare and economic costs of an outbreak are to be reduced. Natural *S. equi* infection in Arabian horse revealed some alteration in hematological and biochemical parameters without effect on hepatic and renal tissues. Moreover, the presence of respiratory acidosis and increased of cTnI could indicate pneumonia secondary to strangles with risk of heart involvement. Treatment with procaine penicillin with benzathine penicillin is useful to relief these alterations toward the healthy values.

## Authors’ Contributions

ANFN and HMED planned the study design. HMED collect data and samples for epidemiological and bacteriological examination. ANFN examine samples for hematological, biochemical, and acid-base evaluation. Both authors drafted, revised, read, and approved the final manuscript.
